# Transcranial photobiomodulation prevents PTSD-like comorbidities in rats experiencing underwater trauma

**DOI:** 10.1038/s41398-021-01389-5

**Published:** 2021-05-05

**Authors:** Yong Li, Yan Dong, Luodan Yang, Lorelei Tucker, Baocheng Yang, Xuemei Zong, Michael R. Hamblin, Quanguang Zhang

**Affiliations:** 1 Department of Neuroscience and Regenerative Medicine, Medical College of Georgia, Augusta University, Augusta, GA USA; 2 Laser Research Centre, Faculty of Health Science, University of Johannesburg, Doornfontein, South Africa; 3 Radiation Biology Research Center, Iran University of Medical Sciences, Tehran, Iran

**Keywords:** Depression, Molecular neuroscience

## Abstract

Maladaptive fear memory processing after a traumatic event is a major contributor to the development of the comorbidities related to posttraumatic stress disorder (PTSD). An intervention to normalize this process could be a first-line treatment to prevent PTSD development. However, little progress has been made in identifying interventions that can prevent trauma survivors from developing PTSD. A treatment that could help trauma survivors cope with traumatic memories and decrease the prevalence of PTSD is thus in high demand. This study was designed to investigate the potential beneficial effects of early photobiomodulation (PBM) interventions to prevent PTSD-like comorbidities in animals. PTSD-like comorbidities in rats were induced by an underwater trauma (UWT) procedure, followed by multiple swimming sessions on later days for memory recall. Immediately after UWT and swimming, rats were restrained with or without PBM treatment (808 nm, 25 mW/cm^2^, 3 J/day). PTSD-like commodities, such as anxiety-like behavior, depression-like behavior, and cognitive dysfunction, were reproduced in UWT-rats. These comorbidities, however, could be prevented by early PBM interventions. By measuring the expression of immediate early genes (IEGs) as neuronal activity markers, we found that PBM treatment differentially regulated *Arc* and *c-fos* expression in the hippocampus and amygdala, two PTSD-related brain regions. Additionally, PBM boosted ATP production and regulated protein expression in the hippocampus following stress. Our results demonstrate that PBM can modulate brain activity in response to traumatic and stressful events and that early PBM intervention can prevent the occurrence of PTSD-like comorbidities in rats.

## Introduction

Posttraumatic stress disorder (PTSD) is a psychiatric disorder that develops after individual experiences or witnesses a traumatic event. The symptoms of PTSD include flashbacks and intrusive memories, avoidance of trauma-associated cues, negative changes in cognition, and hyperarousal^1^. PTSD patients often develop depressive disorders, anxiety disorders, and substance abuse disorders collectively referred to as PTSD comorbidities^2^. The current prevailing treatment strategy to relieve the comorbidities of PTSD relies upon psychotherapy^3^, cognitive behavioral therapy, and pharmacological therapy^4^, primarily using antidepressants and anti-anxiety medications. However, many patients display no therapeutic benefits from antidepressants and anti-anxiety medication, and the frequent and substantial side effects of these drugs are of significant concern^5^. A new treatment that could relieve the comorbidities and symptoms of PTSD is of the utmost necessity.

Following a traumatic event, fear memory develops in three sequential phases: acquisition, storage, and retrieval^6^. Most life-threatening traumatic events are unpredictable, and this uncertainty makes it difficult to apply a treatment that targets the memory acquisition phase. The storage and retrieval phases include memory consolidation and reconsolidation processes and are responsible for storing and updating a long-lasting memory^7^. These phases may be the earliest practical windows for PTSD early intervention^8^. However, these time windows are relatively narrow and are accessible only within the hours following acquisition and retrieval. The strategy of intervening in the immediate aftermath of trauma to improve coping and reduce further distress has previously been proposed and tested and has yielded promising if controversial, results. One such example is the administration of the beta-blocker propranolol^9,10^. A treatment that could target early memory processing windows and minimize the development of PTSD and the mental suffering associated with PTSD comorbidities is in high demand.

Transcranial photobiomodulation (PBM) is an emerging non-invasive physical treatment with promising results in the context of diverse brain disorders^11,12^ such as stroke^13^, traumatic brain injury, cerebral ischemia, Alzheimer’s disease^14^, and Parkinson’s disease^15^. PBM functions via the stimulation of mitochondrial complex IV activity^16^, resulting in increased adenosine triphosphate (ATP) production^17,18^. This boost to energy metabolism promotes cell survival^19^ and stimulates cellular proliferation^20^. Although it has been reported that PBM treatment relieves anxiety and depression^21,22^, it has yet to be determined whether PBM has effects on the anxiety and depression caused by traumatic events, particularly in the context of PTSD. Due to the convenience and safety of PBM application, this study aimed to investigate the potential beneficial effects of early PBM treatment on the prevention of PTSD-like comorbidities in rats.

## Materials and methods

### Animals

Adult male Sprague–Dawley rats (300–500 g) were bred in our campus animal facility. Rats from the same litter were group-housed (2–4 rats per cage) in a temperature- and light-controlled room (23 °C with a light/dark cycle of 0600 hours/1800 hours) with free access to food and water. Litter-mates were randomly assigned and each group had 7–12 rats. The experiments were in compliance with an animal use protocol that was approved by the Institutional Animal Care and Use Committee of Augusta University.

### PTSD model

The PTSD model was induced by underwater trauma^23,24^ with a modified procedure based on a previous report^25^. Multiple swimming reminder sessions followed by immediate restraint were applied because it is well-accepted that both memory reminders and restraint can induce or potentiate PTSD^26,27^. On day 0, rats were pre-exposed to a one-minute swimming session in a water tank (40 × 60 × 40 cm; water height 30 cm; 22 ± 1 °C) to allow rats to encode and remember the context for the following day’s UWT event. On day 1, rats were allowed to swim for 15 s and then underwent UWT for 45 s by restraining rats in a metal cage (25 × 16 × 16 cm). On days 2 through 7, the rats were allowed to swim for 60 s as a trauma reminder. Following UWT and swimming, rats were restrained for 2 min and returned to their home cages.

### PBM treatment

Under restraint in a plastic cone, rats were subjected to one two-minute PBM treatment (808 nm laser, continuous wave, 25 mW/cm^2^, 3 J/day) per day (1^st^ day immediately after UWT) for 7 consecutive days. To deliver PBM, a diode laser (model 808M100, Dragon Lasers, Jilin province, China) was applied by focusing the beam into a 1.5 cm^2^ round spot covering the shaved scalp, as described in our recent work^14^.

### Behavior tests

Rats remained undisturbed for at least one hour before behavior testing. All tests were conducted during the light phase. All behavior tests were recorded and analyzed by “ANY-maze” software (Stoelting Co. Wood Dale, IL). Data collection and analysis were performed blind to the experimenter.

### The elevated plus maze

was used to test anxiety-like behavior^28^. The maze has a 10 cm wide walking track and the length of both the closed and open arms was 110 cm. Each rat was allowed to explore the maze for 5 min. The time a rat spent exploring the open arms was an indicator of anxiety-like behavior. Open arm entries were defined as all four paws entering the open arms for at least one second.

### The forced swimming test

was used to measure depression-like behavior^29^. Rats were tested in a tank (30 × 37 × 56 cm) filled with water to a height of 36 cm and allowed to swim for 5 min. Immobility was defined as a rat not moving its four paws for at least one second.

### The open-field arena test

was used to test mobility and anxiety-like behavior^30^. The arena (50 × 50 × 50 cm) was made of black laminated particle board. The center zone size is 25 × 25 cm. Rats were placed in the arena and allowed to explore for 5 min under 100 lux illumination.

### The Barnes maze

test was used to test hippocampus-dependent spatial memory and was modified by our laboratory, as previously described^14^. Briefly, 3 days of training trials were performed with a maximum trial length of 180 s. On each trial day (one trial/day), the escape latency of each rat from the platform center to the escape chamber was recorded. The probe trial was performed on day 4 to test spatial memory. The escape box was removed, and time spent in the target quadrant where the escape box was previously located was recorded for 90 s. Target quadrant occupancy above 22.5 s is a memory indicator.

### The Y maze

was used for working memory testing^31^. The Y maze was a “Y” shaped maze purchased from San Diego Instruments (San Diego, CA). Distal cues were decorated over each arm of the maze to facilitate rats differentiating the three arms by remembering the three different visible cues. Rats were allowed to explore the arms for 5 min. The percentage of spontaneous alternation was defined as consecutive entries in 3 different arms, divided by the number of possible alternations (total arm entries minus 2).

### RT-PCR

The brain was immediately harvested under heavy anesthesia. Coronal brain slices (500 µm thickness) were made on a vibrating slicer. The dorsal hippocampus and amygdala (Bregma −2.3 mm to −2.8 mm) were isolated and homogenized as previously described^32,33^. RNA from brain tissue was isolated using an RNeasy Mini kit (QIAGEN Sciences Inc., Germanton, MD) and then digested with DNase to remove contamination with residual genomic DNA. A reverse transcription kit SuperScript III First-Strand Synthesis System (Invitrogen, Carlsbad, CA) was used to synthesize cDNA. The regents for qPCR were from the SsoAdvanced Universal SYBR green super mix (Bio-Rad, Hercules, CA). Gene expression was calculated by the ΔCt method as described previously^34^. The primer sequences are listed as below as previously reported^35^.

Arc forward: GAATTTGCTATGCCAACTCACGGG

Arc reverse: AGTCATGGAGCCGAAGTCTGCTTT

BDNF forward: TGTCTCTGCTTCCTTCCCACAGTT

BDNF reverse: TGGACGTTTGCTTCTTTCATGGGC

c-fos forward: ACAGCCTTTCCTACTACCATTCCC

c-fos reverse: CTGCACAAAGCCAAACTCACCTGT

GAPDH forward: AGAGACAGCCGCATCTTCTTG

GAPDH reverse: GGTAACCAGGCGTCCGATAC

### ATP detection

Rats were anesthetized by inhalation of isoflurane followed by cardiac perfusion with ice-cold PBS. The hippocampus was collected quickly, and the dorsal portion of the hippocampus was homogenized in 300 µl ice-cold lysis buffer (50 mM HEPES, pH 7.4, 150 mM NaCl, 12 mM beta-glycerophosphate, 1% Triton) with protease inhibitor cocktail (Sigma-Aldrich, St. Louis, MO). The lysate was then sonicated for 30 s and centrifuged at 12000 rpm for 10 min at 4 °C. The supernatant was used for ATP detection (Firefly Luciferase Bioluminescence Assay, Invitrogen). Protein concentration was measured by a BCA protein assay kit (Themo Fisher Scientific, Waltham, MA), and 5 µg of total protein was assayed for measuring ATP. ATP concentration was quantified according to the standard curve.

### Plasma corticosterone

Plasma was collected rapidly from arterial blood after the right auricle was cut open for cardiac perfusion. Blood was collected in a 1.5 ml tube containing 25 mg EDTA and then centrifuged at 2000 rpm for 10 min. The supernatant plasma (0.5 µl/well) was assayed via a corticosterone kit (EIACORT, Invitrogen). Corticosterone was quantified according to the standard curve.

### Mass spectrometry

The sample aliquots (100 µg protein/sample) prepared as above for measuring ATP in the hippocampus were sent to our proteomic core facilities for mass spectrometry analysis. Briefly, the proteins were precipitated by adding trichloroacetic acid and then were digested by trypsin. After digestion, the peptide was subjected to LC-MS/MS analysis as described^36^. The calculation of the Z score and the protein classification were analyzed using the PANTHER Classification System^37^.

### Western blot

Aliquots of hippocampus lysis prepared for mass spectrometry were incubated with Laemmli sample buffer (Bio-Rad) at 95 °C for 5 min to denature the proteins. Then, proteins (20 µg/lane) were separated by a 4-20% precast polyacrylamide gel (Bio-Rad) and transferred onto a PVDF membrane. After blocking, the membrane was incubated with primary antibodies (PPME1; # A304-762A-T, Bethyl Laboratories, Inc.,Montgomery, TX) (TIF1β; # 4123 T, Cell Signaling Technology, Danvers, MA) (MIC60; # A305-024A-T, Bethyl Laboratories, Inc.) (GRAP1; # 398198, Santa Cruz Biotechnology, Inc. Dallas, TX) (β-tubulin; # 480011, Thermo Scientific). After incubating with HRP-conjugated secondary antibodies, the protein bands were visualized by incubating the membrane with chemiluminescence detection solution (Thermo Scientific). Images were captured by the ImageQuant LAS 4000 system (GE Healthcare). The membranes were then re-probed with different primary antibodies after stripping (stripping buffer: 50 mM glycine and 0.1% SDS, pH = 2). The protein band signal was quantified with ImageJ software (NIH, Bethesda, MD). Band-free background intensity was first subtracted from each band intensity, and then band intensities were normalized to β-tubulin signal. Protein bands of littermates of three animals were quantified together to minimize individual differences. The protein expression level of each ctrl rat was normalized as 100%. The relative fold changes of protein expression in the UWT and PBM rats were individually plotted.

### Statistical analysis

All data were presented as mean ± SE. Statistical comparisons between multiple groups were analyzed with one-way ANOVA using SigmaStat 3.5 software. After ANOVA, Tukey post hoc tests were used for pairwise comparisons between different groups. The difference in expressions of IEGs between PBM-group and Restrain-group was analyzed via student t-test (two tailed). A level of *P* < 0.05 was considered statistically significant. * denotes *P* < 0.05 for a significant treatment effect in the PBM-group compared with the UWT-group; # denotes *P* < 0.05 for the existence of a PTSD-like phenotype in the UWT-group compared with the ctrl-group.

## Results

### Early PBM interventions prevented PTSD-like comorbidities

**Fig. 1:**
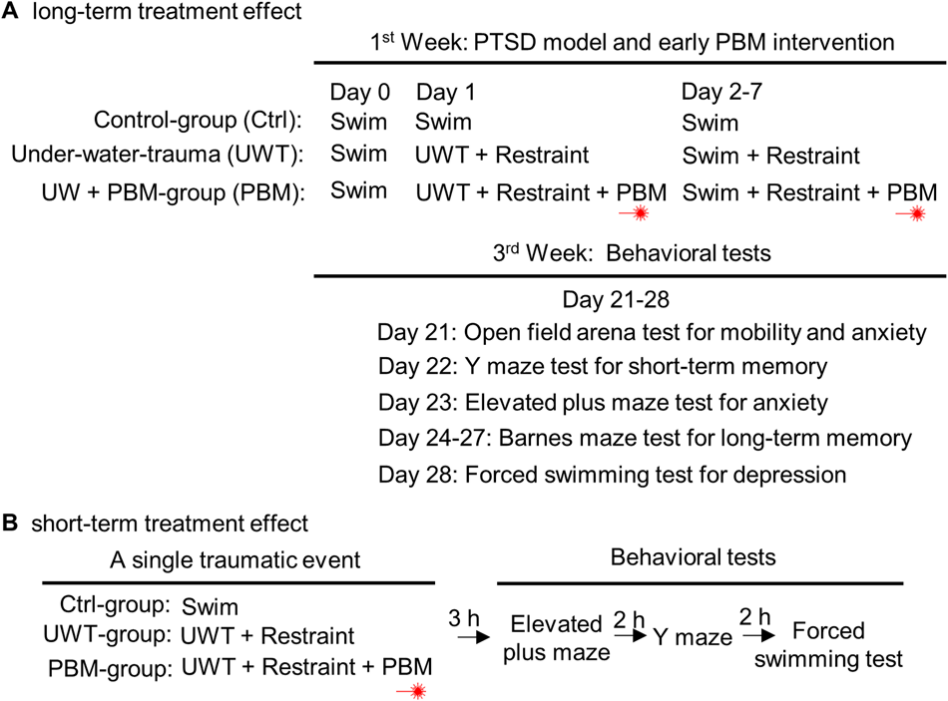
Experiment design paradigm. **A** UWT-group rats were restrained immediately following UWT at day 1. From day 2 to day 7, rats were allowed to swim for 1 min/day as a reminder session. Rats were immediately restrained (2 min) following UWT and swimming sessions. During restraint, rats who received PBM treatment (2 min) were assigned as PBM-group, and rats that did not receive PBM treatment were assigned as UWT-group. In total, each PBM-group rat received 7 doses of PBM treatment. Ctrl-group rats were allowed to swim freely from day 0 to day 7. All rats remained undisturbed during days 8–20. A battery of behavioral tests was performed, beginning on day 21. **B** Rats underwent the same UWT event and PBM treatment as the above experimental design on day 1. Three hours later, a battery of behavioral tests for PTSD-like comorbidities was initiated.

We first assigned three groups of rats (Fig. 1A) for exploring the long-term treatment effect from PBM: (I) ctrl-group rats were subjected to swimming on days 0–7 without restraint after swimming; (II) UWT-group rats were subjected to UWT at day 1 and swimming reminder sessions on day 2–7, followed by immediate restraint for 2 min after removal from the water tank; (III) PBM-group rats underwent the same procedure as the UWT-group but received PBM treatment (one dose per day for 7 days) under restraint. A battery of behavior test was performed three weeks after UWT. This experimental design allowed us to evaluate the occurrence of PTSD-like comorbidities (by statistical comparison between the ctrl-group and UWT-group) and the long-term treatment effect from PBM (by statistical comparison between the UWT-group and PBM-group). We also assigned three additional groups of rats (Fig. 1B) to explore the short-term treatment effect from PBM. The rats in these three groups underwent the same procedure as previous groups, but the behavioral tests were started at 3 h after UWT.

**Fig. 2:**
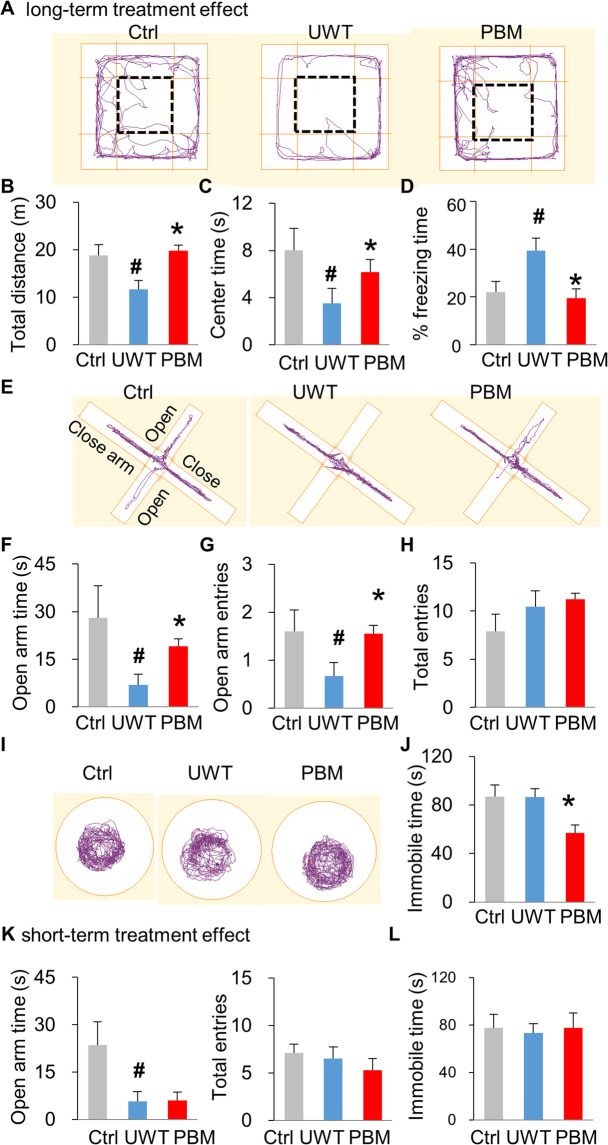
PTSD-like comorbidities were prevented by PBM early interventions. **A–D** PBM restores exploratory motor activity deficits in the open field arena test. **A** The apparatus and the representative exploration track for rats. The rectangle with a bold dashed line denotes the center zone. Three weeks post UWT, rats in UWT-group exhibited a significant reduction of total traveling distance in both the whole arena (**B**) and center zone (**C**), compared with the ctrl-group. PBM treatment restored exploratory activity for both the whole arena and center zone. **D** During the open field arena test, a higher percent of freezing was observed in UW-group rats but was reversed by PBM treatment. **E**–**H** PBM relieves anxiety-like behavior in the elevated plus maze test. **E** The apparatus for elevated plus maze. Rats in UWT-group spent less time exploring the open arms compared with ctrl-group rats. **f** PBM treatment increased the time visiting open arms compared to UWT-group rats. **g** Open arm entries mirrored the trend observed in time spent in open arms. **H** As a measurement control, all three groups had similar total arm entries. **I**, **J** PBM alleviates depression-like behavior in the forced swimming test. **I** The apparatus for forced swimming test. **J** PBM decreased the immobile time in PBM-group compared with the other two groups. *n* = 10, 9, 9 for ctrl, UWT, PBM-group, respectively. **K**, **L** A single dose of PBM treatment has no short-term treatment effect on either the elevated plus maze (**K**) and forced swimming test (**L**). To be noted, the anxiety-like, but not the depression-like behavior, was observed at 3 h post UWT. *n* = 8 each for ctrl-, UWT-, or PBM-group. * *P* < 0.05 versus UWT-group; #*P* < 0.05 versus ctrl-group.

Three weeks after UWT, rats were first subjected to the open field arena test (Fig. 2A). Rats in UWT-group (11.67 ± 1.88 meters) traveled less distance (*P* = 0.030, ctrl vs. UWT) compared with those in ctrl-group (18.82 ± 2.27 meters). In contrast, PBM-group rats (19.82 ± 1.19 meters) had a longer travel distance than the UWT group (*P* = 0.002, PBM vs. UWT), similar to that of the ctrl-group (Fig. 2B). Another parameter of the open field test, the time rats spent visiting the center area, was used to indicate the absence of anxiety-like behavior^30^, with results following a similar trend. Rats in UWT-group spent less time visiting the center area compared with ctrl-group rats (*P* = 0.038, ctrl vs. UWT), which suggests the presence of an anxiety-like phenotype in UWT-group. This anxiety-like behavioral phenotype could be prevented by PBM treatment (*P* = 0.034, PBM vs. UWT) (Fig. 2C). Interestingly, we found that UWT-group rats had a significantly longer freezing time, another parameter for measuring anxiety-like behavior, compared with the other two groups (Fig. 2D)^38,39^. These results suggest that UWT induced an anxiety-like phenotype in the UWT-group and that PBM-treatment could prevent this anxiety-like behavior.

To further confirm the effects of PBM on anxiety-like behavior, rats underwent the elevated plus maze test (Fig. 2E), a behavioral test that measures anxiety-like behavior. UWT-group rats (7.02 ± 3.26 s) spent less time visiting the open arms, a sign of decreased anxiety-like behavior, compared with those in ctrl-group (28.09 ± 10.15 s) (*P* = 0.046, ctrl vs. UWT). Rats in the PBM-group displayed a treatment effect and explored the open arm for a greater duration (19.14 ± 2.26 s) (*P* = 0.006, PBM vs. UWT) (Fig. 2F). Quantification of the number of open arm entries (Fig. 2G) revealed results that mirrored those of the time spent exploring the open arms. As a control, the three groups made a similar number of total entries (Fig. 2H) to both the open arms and closed arms, demonstrating a comparable degree of exploratory behavior.

Depression is another comorbidity of PTSD. Rats were tested in the forced swimming test (Fig. 2I) that measures immobile time as an indicator for depression-like behavior. When compared with ctrl-group (86.95 ± 9.38 s), however, we did not observe depression-like behavior in UWT-group (86.58 ± 6.89 s), while rats in the PBM-group (56.86 ± 6.66 s) exhibited significantly less immobile time compared with the other two groups (*P* = 0.011, PBM vs. UWT) (Fig. 2J). This result suggests that PBM treatment could relieve basal levels of depression-like behavior present under normal conditions. Although a long-term treatment effect of PBM was evidenced from the above findings, PBM did not exhibit a short-term treatment effect on either the elevated plus maze testing for anxiety-like behavior (Fig. 2K) or the forced swimming testing for depression-like behavior (Fig. 2L).

### Cognitive dysfunction was prevented by PBM treatment

**Fig. 3:**
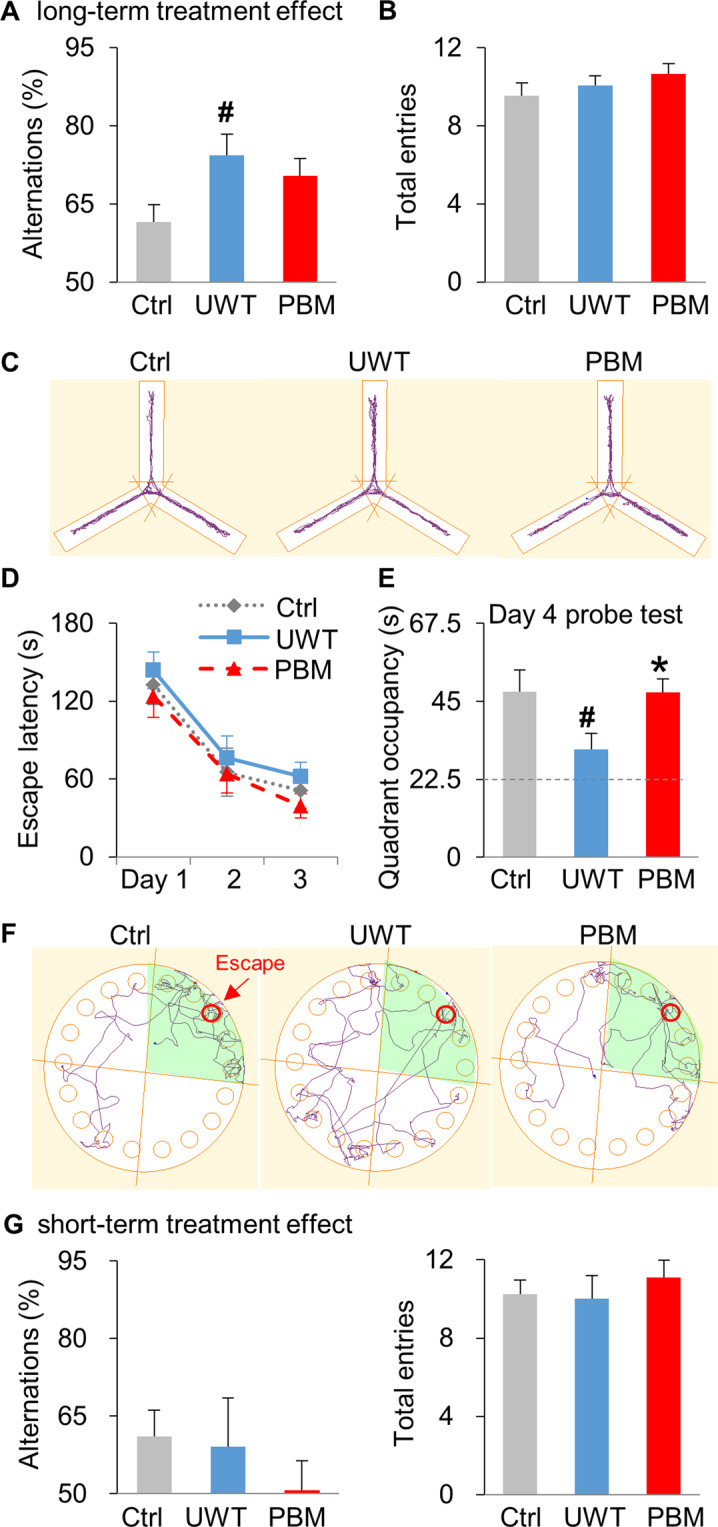
Cognitive dysfunction was prevented by PBM early interventions. **A–C** UWT enhances working memory in the Y maze test, and PBM has no treatment effect. **A** Compared with the ctrl-group, UWT-group displayed better working memory, as shown by a higher percentage of alternation. **B** As a behavioral measurement control, all three groups had a similar number of total entries. **C** The apparatus and the representative exploration tracks for each group during Y maze testing. **D–F** PBM prevents long-term memory impairment in the Barnes maze test. **D** All three groups exhibited the same learning curve during the three days of training sessions (one trial/ day). **E** During the probe test session (90 s duration) at day 4, rats in UWT-group exhibited significantly decreased preference (22.5 s is random chance as labeled with dash line) for exploring the target quadrant where the escape chamber was previously located, compared with the other two groups. **F** The apparatus and the representative tracks on the probe test (day 4) for all three groups. Target quadrant is shaded, and the red circle denotes the location for the escape chamber. *n* = 10, 9, 9 for ctrl, UWT, PBM-group, respectively. **G** A single dose of PBM treatment has no short-term treatment effect on Y maze testing. *n* = 8 each for ctrl, UWT, or PBM-group. * *P* < 0.05 versus UWT-group; # *P* < 0.05 versus ctrl-group.

Cognitive dysfunction is a comorbidity that often co-exists with PTSD symptoms in patients^40^. To investigate whether the early PBM treatment has a long-term effect on memories, we first tested short-term working memory using the Y maze test (Fig. 3A). Interestingly, rats in UWT-group (74.33 ± 4.09% alternation) had a higher percentage of spontaneous alternation than those in ctrl-group (61.57 ± 3.36% alternation), indicating better working memory in the UWT-group (*P* = 0.029, ctrl vs. UWT). PBM treatment did not affect this improved working memory, and the percentage of spontaneous alternation observed in PBM-group rats was 70.39 ± 3.36%. As a behavior measurement control for the Y maze test, the three groups each made a similar number of total entries to the three arms (Fig. 3B, C), which indicates a similar total number of exploring choices made between the three groups. Next, long-term spatial memory was tested in the Barnes maze. The three groups displayed a similar learning curve to one another during the training trials over the first three days of testing (Fig. 3D). When testing spatial memory during the probe trial on day 4, however, UWT-group rats (31.22 ± 4.59 s) exhibited a significant long-term memory deficit compared to the ctrl-group. Meanwhile, rats in the ctrl-group (47.81 ± 6.24 s) and PBM-group (47.59 ± 3.95 s) displayed a higher preference for exploring the target quadrant (*P* = 0.041, ctrl vs. UWT; *P* = 0.013, PBM vs. UWT) (Fig. 3E, F). This result indicates that early PBM treatment prevents the decline in long-term memory associated with PTSD comorbidity. A single PBM treatment dose has no short-term treatment effect on Y maze testing for working memory (Fig. 3G).

### Expression of immediate early genes (IEGs) in the hippocampus and amygdala were altered differentially by PBM

Reduced volume and activity in the hippocampus and amygdala hyperactivity are factors implicated in PTSD psychopathology^41^. To investigate whether PBM affects the activity in these two PTSD-related brain regions, we focused on the expression of immediate early genes (IEGs), including *Arc* and *c-fos*. IEGs are the first group of genes expressed within minutes or hours after synaptic and neuronal activation triggered by external behavioral activities^35^. Expressional changes of IEG transcription in a specific brain region reflect a stimulation or inhibition of the neuronal activity in this brain region. Often exploited for its role as a neuronal activity marker, *Arc* is an activity-regulated cytoskeleton-associated protein essential for supporting the structure changes undergirding synaptic plasticity and the memory consolidation process^42^. As an inducible transcription factor, rapid and transient expression of *c-fos* is associated with the initial steps of transcriptional regulation that supports long-term memory storage in different learning tasks^43^.

**Fig. 4:**
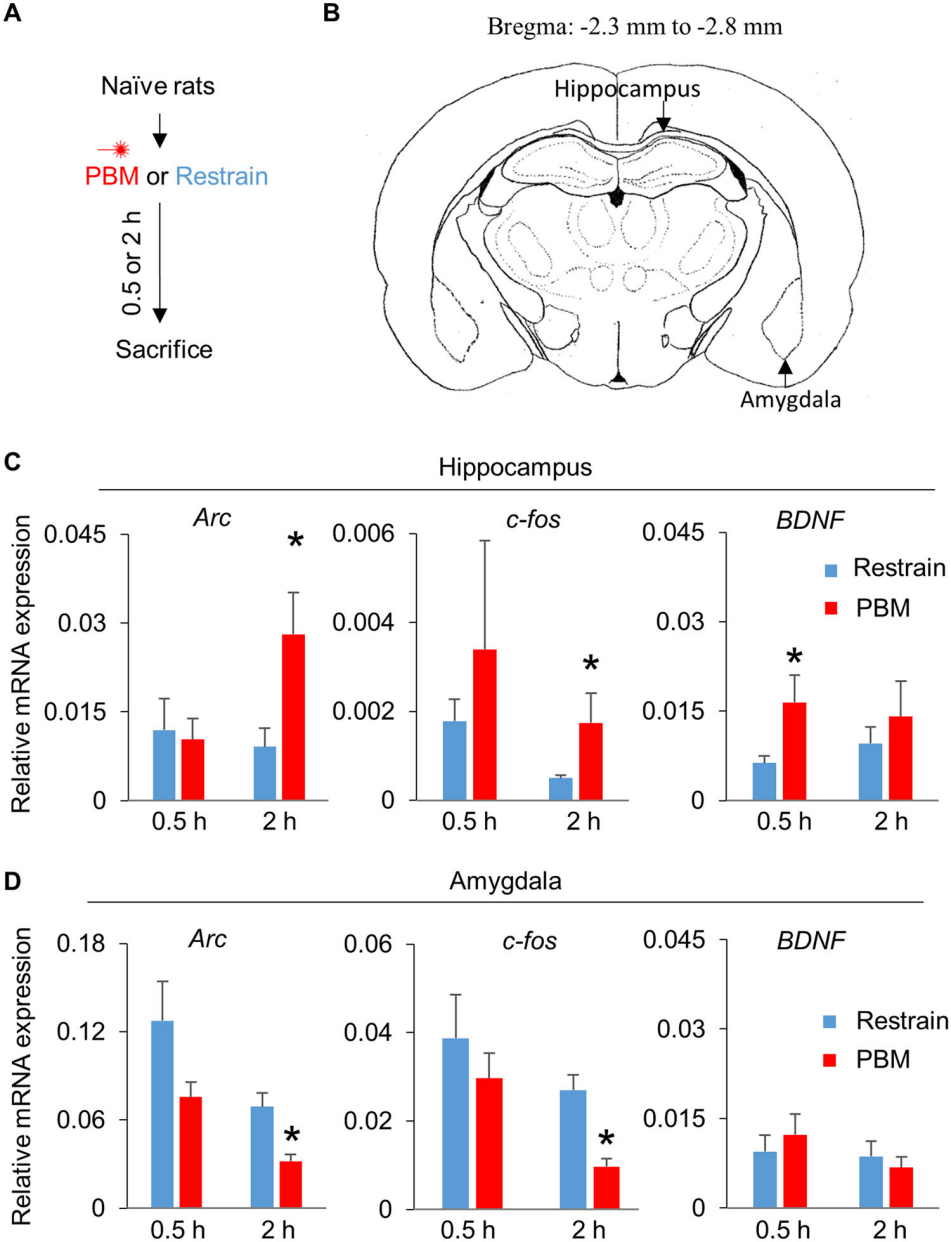
PBM induced *Arc*, *c-fos*, and *BNDF* transcription in the hippocampus, and repressed *Arc*, *c-fos* expression in the amygdala. **A** Naive rats were restrained along with or without PBM, and then were sacrificed at 0.5 h and 2 h post PBM. **B** Brain atlas for collecting hippocampus and amygdala tissue. **C** PBM treatment increased *Arc*, *c-fos*, and *BNDF* transcription in the hippocampus. **D** PBM inhibited *Arc* and *c-fos* expression in the amygdala. *n* = 7, 7 for Res, PBM-group at 0.5 h, respectively; *n* = 7, 6 for Res, PBM-group at 2 h, respectively. *P < 0.05 versus Restrain-group.

Naive rats were subjected to restraint for two minutes with or without PBM treatment (Fig. 4A), and the hippocampus and amygdala (Fig. 4B) were collected at 0.5 h and 2 h post-treatment. Intron-exon junctions were selected as primer binding sites for detecting newly synthesized RNA transcripts. Interestingly, compared with the restrain-group, rats in PBM-group rats had higher levels of newly formed *Arc* (*P* = 0.034 at 2 h)*, c-fos* (*P* = 0.047 at 2 h), and *BDNF* (*P* = 0.032 at 0.5 h) mRNA transcripts in the hippocampus (Fig. 4C), but less *Arc* (*P* = 0.006 at 2 h) and *c-fos* (*P* = 0.002 at 2 h) transcripts in the amygdala (Fig. 4D) following restraint.

### PBM boosts ATP production in the hippocampus but has no effects on corticosterone

**Fig. 5:**
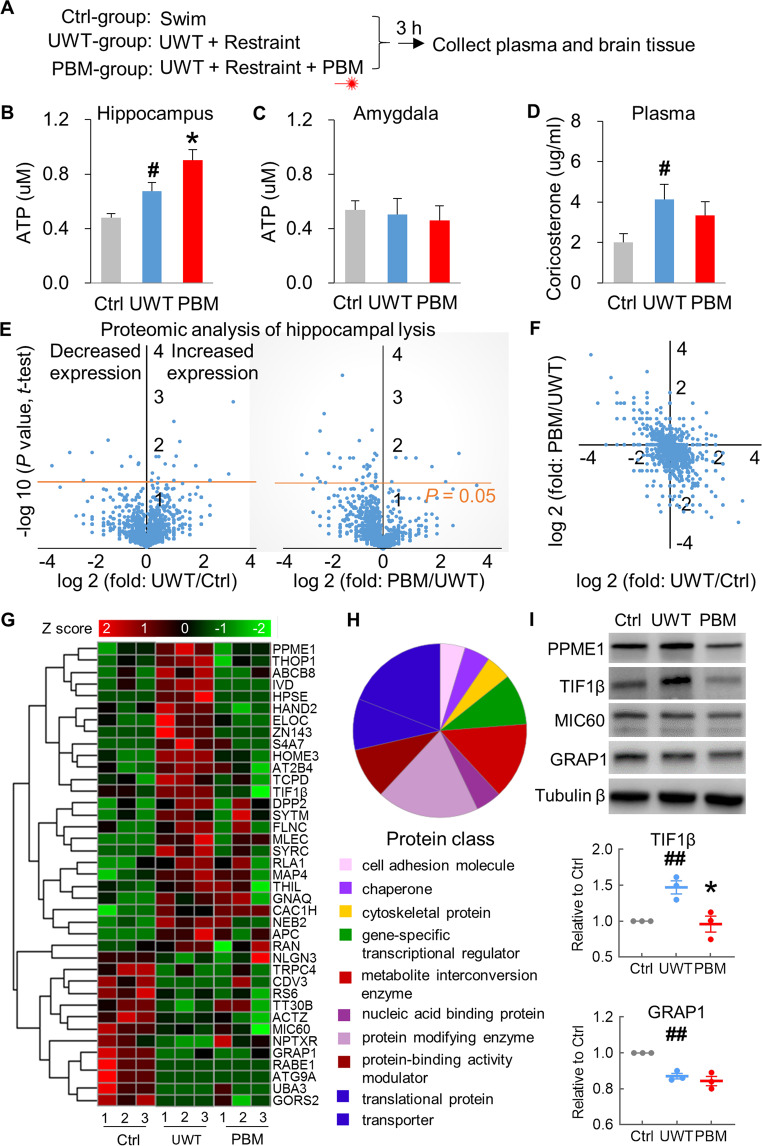
PBM boosted ATP production and differentially regulated protein expression in the hippocampus. **A** After UWT, rats were restrained together with or without receiving PBM treatment. Rats were sacrificed at 3 h post PBM treatment. **B** Acute stress-induced higher ATP production in UWT-group. PBM further increased ATP in the hippocampus of PBM-group rats. **C** Amygdala ATP content was unchanged, and PBM had no treatment effects. **D** Plasma corticosterone was increased in UWT-group, compared with the ctrl-group. PBM treatment had no treatment effects. *n* = 9, 12, 12 for ctrl, UWT, PBM-group, respectively. * *P* < 0.05 versus UWT-group; #*P* < 0.05 versus ctrl-group. **E** The volcano plot of proteins identified by mass spectrometry (*n* = 3 each group). Left panel (UWT/ctrl): the dots above the orange line (*P* = 0.05) are the proteins that were significantly decreased (left side of the Y-axis) or increased (right side) in the UWT-group as compared with the ctrl-group (UWT/ctrl). Right panel (PBM/UWT): more dots on the left side of the Y-axis than the right side indicates that PBM down-regulated the proteins that were over-expressed by UWT. **F** A two-dimension plot for the ratio of UWT/ctrl and PBM/UWT. **G** The heat map for the expression profiles of 39 proteins identified with significantly differential expression (UWT vs. ctrl). Each column represents an individual rat belonging to a certain group. **H** The proportion and classification of the 39 identified proteins. **I** Confirmation of the mass spectrometry results with Western blot. Top panel: Representative protein expression files from the rats in ctrl, UWT, and PBM-group. Middle and bottom panel: the quantification of Western blot results for TIF1β and TRAP1. * *P* < 0.05 versus UWT-group; ## *P* < 0.01 versus ctrl-group.

We then investigated whether PBM affected ATP production, a well-accepted target of PBM^44^. Rats were subjected to UWT followed by restraint (UWT-group), and the hippocampus and amygdala were collected at 3 h post-UWT (Fig. 5A). PBM-group rats received treatment during restraint. Ctrl-group rats swam for 1 min but did not receive UWT and restraint. Rats in the UWT-group exhibited higher ATP levels in the hippocampus compared with ctrl-group (*P* = 0.029), and lower ATP content compared with PBM-group (*P* = 0.040) (Fig. 5B). ATP production in the amygdala remained unchanged following UWT, and PBM had no treatment effects (Fig. 5C). This result suggests that PBM could boost ATP production for energy consumption in response to trauma and stressful events. It was possible that, as stressors, UWT and restraint may induce stress hormone release, which may, in turn, stimulate energy metabolism and ATP production. As seen in Fig. 5D, plasma corticosterone increased following UWT and restraint (*P* = 0.036, UWT vs. ctrl). However, this increase in plasma corticosterone was unaffected by PBM.

Since PBM is well-established to boost hippocampal ATP production, we performed proteomic analysis on hippocampal lysis (*n* = 3 each for ctrl-, PBM- and UWT-group, totally 9 samples) to further explore the molecular mechanisms underlying the beneficial effects of PBM. The mass spectrometry results were listed in Supplementary Table 1. 39 proteins have been identified to decrease or increase expression in the UWT-group (Fig. 5E left panel) compared the ctrl-group. These protein candidates can be considered differentially expressed proteins induced explicitly by a severe UWT event. Interestingly, by comparing the results of the UWT-group with the PBM-group (Fig. 5E right panel), we found that PBM contributes more to an inhibitory effect on protein expression than a stimulating effect, as more dots were observed above the significance line (orange, *P* = 0.05) on the left side (43 proteins) of Y the axis than the right side (7 proteins). These 50 proteins can be considered differentially expressed protein candidates induced explicitly by a single PBM treatment dose. We have also presented a two-dimensional plot (Fig. 5F) that lists the ratios of UWT/ctrl and PBM/UWT for proteins detected by mass spectrometry (4255 proteins), as well as a heat map (Fig. 5G) that includes the names and expression profiles of 39 proteins that were differentially expressed after UWT. These results suggest that PBM treatment downregulates the expression of proteins that are upregulated by UWT. The proportions and classifications of the 39 identified proteins are displayed in Fig. 5H. These, listed in order from highest proportion to lowest, represent protein modifying enzymes, transporters, metabolite interconversion enzymes, translational proteins, protein-binding activity modulators, and gene-specific transcriptional regulators, and more. To confirm the above proteomic findings, we selected four proteins from the list in Fig. 5G to analyze via Western blot. Among these four proteins, PPME1 and TIF1β were upregulated by UWT, while MIC60 and GRAP1 were downregulated by UWT. As shown in the top panel of Fig. 5I, our Western blot results are consistent with the mass spectrometry findings. PBM treatment downregulates the expression of TIF1β that is upregulated by UWT (Fig. 5I middle panel; *P* = 0.006, UWT vs. ctrl; *P* = 0.024, PBM vs. UWT). However, PBM does not affect the downregulated expression of GRAP1 induced by UWT (Fig. 5I bottom panel; *P* = 0.001, UWT vs. ctrl; *P* = 0.426, PBM vs. UWT).

## Discussion

Here, we report that early PBM interventions prevented PTSD-like comorbidities (Fig. 2), including anxiety-like behavior, depression-like behavior, and cognitive deficits in a UWT rat model of PTSD (Fig. 3). Differential changes in the patterns of IEG expressions (Fig. 4) and ATP production (Fig. 5) in the hippocampus and amygdala after PBM treatment suggest that PBM modulates neuronal activities in these two critical PTSD-related brain regions. Since we applied PBM as early intervention during the consolidation^45^ and reconsolidation^46^ windows of the trauma-associated memories, whether delayed PBM treatment applied outside these windows can generate the same treatment effect is as yet unknown and warrants further investigation.

It is important to note that PBM alleviated UWT-induced memory deficits in the Barnes maze test (Fig. 3E), a long-term spatial memory test that depends on hippocampal function^47^. This suggests that the hippocampus benefits from PBM treatment. Our RT-PCR data (Fig. 4) supports this conclusion, as we observed increases in the expressions of IEGs in the hippocampus. Intriguingly, the expression of IEGs in the amygdala was decreased. It is well accepted that hyperactivity of the amygdala plays a causal role in the experience of negative emotions such as fear, anxiety, and distress^48^. The inhibitory effect of PBM in the amygdala could partially explain why PBM relieved PTSD-like comorbidities.

While UWT rats displayed deficits compared to the ctrl-group in long-term memory during Barnes maze testing (Fig. 3E), UWT rats exhibited better short-term memory during Y maze testing (Fig. 3A). It is still under debate whether memory is enhanced or impaired under the influence of stress, as differences in the source, duration, intensity, and timing of a stressor generate conflicting outcomes regarding memory phase and learning type^49^. In this study, stressors were applied two-weeks before memory tests and WT-group rats exhibited higher basal levels of anxiety-like behavior in the open field test (Fig. 2C) and elevated plus maze (Fig. 2F) before memory testing. During memory testing, the emotional state of an animal, such as anxiety and depression, may interfere with memory consolidation, a process that converts a short-term memory into a long-term memory^7^. The improvement in long-term memory induced by PBM treatment suggests that PBM can protect the memory consolidation process.

It is possible that PBM treatment applied immediately after the UWT event may inhibit the short or long term memory of the traumatic event. In this case, the rat would not remember the traumatic experience because the memory was somehow “lost”. This explanation leaves a critical concern regarding whether PBM is enhancing or dampening the traumatic memory. To address this concern, three additional groups of rats were tested for an acute treatment effect from a single dose of PBM treatment (Figs. 1B and 2K). We found that PBM can decrease anxiety-like behavior at three weeks (Fig. 2F), but not at three hours (Fig. 2K), after the UWT event. This finding suggests that at least three hours after the UWT event, PBM-treated rats have an intact long-term memory about the traumatic event. We have also tested PBM on another PTSD model with fear conditioning training that pairs the tone with a foot shock in a specific context (unpublished data). We found that PBM treatment applied immediately after the fear conditioning event can protect the traumatic event’s long-term contextual memory. The PBM-treated animals present less PTSD comorbidities in both PTSD models because a precise contextual memory contributes to context discrimination and fear extinction.

Consistent with other reports that PBM increases *BDNF* expression^50,51^, we observed that, at 0.5 h post restraint, hippocampal *BNDF* expression in PBM-treated rats was nearly three-fold (Fig. 4C) higher than the control group. This substantial increase in *BDNF* suggests that PBM treatment could produce other beneficial effects, such as modulation of neural plasticity and learning^52^. It worth noting that hippocampal BNDF is a driving factor for the facilitation of fear extinction^53^, a process which weakens a fear response to a conditioned cue^54^. These results are highly clinically relevant, as PTSD patients are well known to have deficits in extinction learning^55^. It has been previously reported that PBM facilitates fear extinction^56^; therefore PBM may also be beneficial for several fear-related disorders, such as phobias, panic disorder, etc.

ATP is the primary source of cellular energy for the brain, which consumes 20% of the ATP produced in the body^57^. We observed an increase in ATP production in the hippocampus after PBM treatment (Fig. 5B). It has been reported that metabolic agents that enhance ATP production can improve cognitive functioning^58^, likely in part because ATP plays a critical role in maintaining long-term potentiation (LTP), an electrophysiological process underlying memory formation^59^. In this manner, increased ATP output could be another underlying mechanism for the cognitive improvements observed in PBM-treated rats.

In addition to the above ATP findings, proteomic analysis revealed that PBM treatment downregulated hippocampal expression of proteins that were upregulated by the UWT event (Fig. 5E–H), including protein modifying enzymes, and transporters, metabolite interconversion enzymes, translational proteins, and protein-binding activity modulators. This result suggests that PBM can generate a broad effect on the regulation of protein expression. Beyond classically accepted mitochondrial proteins affected by PBM, we identified ATP-binding cassette sub-family B member 8 (ABCB8) as a target of PBM. Interestingly, transcription elongation factor B polypeptide 1 (ELOC) and transcription intermediary factor 1-beta (TIF1β) were also identified in our mass spectrometry result as being affected by PBM. As gene-specific transcriptional regulators^60,61^, changes in these two proteins’ expressions are expected to generate a long-term and profound effect on regulating gene expression in the hippocampus. In line with a recent study that reported optogenetic stimulation of the hippocampus during the fear conditioning event can prevent the development of PTSD-like memory impairments^62^, these findings highlight that activation of hippocampus during memory processing phases alleviates the development of PTSD-like comorbidities.

Underwater trauma, swimming, and transient restraint are all potent stressors. Acute stress causes a rapid release of the stress hormones norepinephrine and glucocorticoids, both of which bind to hippocampal receptors and facilitate memory consolidation^63,64^. Alterations in hippocampal metabolism during stress are the key to understanding the effects of stress hormones on hippocampal-dependent memory formation. Norepinephrine promotes additional glucose metabolism within minutes, while hours into the stress response process, glucocorticoids act to suppress metabolism^65^. In our results, PBM had no significant effects on glucocorticoid levels (corticosterone in rats) 3 h after UWT (Fig. 5D). Whether PBM has acute effects on norepinephrine in the minutes following UWT is unknown.

In summary, our results suggest that PBM has the potential to be a non-invasive treatment to prevent the occurrence of PTSD-like comorbidities if applied at early intervention windows. However, the optimal PBM parameters for human treatment are yet to be determined. Since human skulls are much thicker than rat skulls, the limited penetration depth of PBM in the human brain is a concern. We propose four potential ways to circumvent this difficulty: increasing laser output power for deeper light penetration; using multiple light sources from different directions that converge at one central brain region; applying PBM to the thinnest part of the human skull, the temporal bone (thickness: frontal, 5.7 mm; temporal, 3.4 mm; occipital, 7.1 mm; parietal, 5.6 mm)^66^ which covers temporal lobe where the hippocampus and amygdala are located; skull trepanning and laser fiber implantation if necessary. In this study, PBM was applied to the rat’s entire brain. Although we confirmed that the hippocampus responded to PBM treatment, whether and how other brain areas react to PBM treatment has not yet been tested. PBM is an emerging therapy for the treatment of psychiatric diseases, and future clinical investigation may establish whether PBM could be a front-line approach for the prevention and treatment of PTSD, offering patients the hope of a better life.

## Supplementary information

Supplementary table 1

## Data Availability

The data that supports the findings of this study are available from the corresponding author upon request.
